# Unveiling Spatial Associations between COVID-19 Severe Health Index, Racial/Ethnic Composition, and Community Factors in the United States

**DOI:** 10.3390/ijerph20176643

**Published:** 2023-08-24

**Authors:** Ruaa Al Juboori, Divya S. Subramaniam, Leslie Hinyard, J. S. Onésimo Sandoval

**Affiliations:** 1 School of Applied Sciences, The University of Mississippi, Oxford, MS 38677, USA; 2 Department of Health and Clinical Outcomes Research, Advanced HEAlth Data (AHEAD) Institute, Saint Louis University, St. Louis, MO 63103, USA; divya.subramaniam@health.slu.edu (D.S.S.); leslie.hinyard@health.slu.edu (L.H.); 3 Department of Sociology and Anthropology, Saint Louis University, St. Louis, MO 63103, USA; ness.sandoval@slu.edu

**Keywords:** racial disparities, chronic disease inequalities, comorbidity index, pandemic, spatial analysis, disease burden, COVID-19

## Abstract

There are limited efforts to incorporate different predisposing factors into prediction models that account for population racial/ethnic composition in exploring the burden of high COVID-19 Severe Health Risk Index (COVID-19 SHRI) scores. This index quantifies the risk of severe COVID-19 symptoms among a county’s population depending on the presence of some chronic conditions. These conditions, as identified by the Centers for Disease Control and Prevention (CDC), include Chronic Obstructive Pulmonary Disease (COPD), heart disease, high blood pressure, diabetes, and obesity. Therefore, the objectives of this study were (1) to investigate potential population risk factors preceding the COVID-19 pandemic that are associated with the COVID-19 SHRI utilizing non-spatial regression models and (2) to evaluate the performance of spatial regression models in comparison to non-spatial regression models. The study used county-level data for 3107 United States counties, utilizing publicly available datasets. Analyses were carried out by constructing spatial and non-spatial regression models. Majority White and majority Hispanic counties showed lower COVID-19 SHRI scores when compared to majority Black counties. Counties with an older population, low income, high smoking, high reported insufficient sleep, and a high percentage of preventable hospitalizations had higher COVID-19 SHRI scores. Counties with better health access and internet coverage had lower COVID-19 SHRI scores. This study helped to identify the county-level characteristics of risk populations to help guide resource allocation efforts. Also, the study showed that the spatial regression models outperformed the non-spatial regression models. Racial/ethnic inequalities were associated with disparities in the burden of high COVID-19 SHRI scores. Therefore, addressing these factors is essential to decrease inequalities in health outcomes. This work provides the baseline typology to further explore many social, health, economic, and political factors that contribute to different health outcomes.

## 1. Introduction

The presence of pre-existing burdens of chronic diseases in the US significantly amplifies the severity of COVID-19 complications. These chronic diseases are closely linked to inequalities in social determinants of health [1]. Presently, there is a substantial population of non-elderly Americans, ranging from 50 to 129 million individuals (19% to 50%), who have comorbidities or a pre-existing health condition [2]. Many studies have consistently demonstrated a clear correlation between the presence of comorbidities and worse rates of COVID-19 infections, hospitalizations, and death throughout the COVID-19 pandemic [3,4,5,6,7,8,9,10,11,12,13]. Research investigating the biological basis of preexisting comorbidities and their impact on COVID-19 clinical outcomes has identified two key assumptions for this association: (1) the prolonged inflammatory process and (2) the dysregulated response of the immune system that accompanies the chronic conditions. These factors led to a poor prognosis in patients infected with COVID-19 [4]. Previous systematic reviews and meta-analyses showed that common comorbidities such as diabetes [5], hypertension [6], chronic obstructive pulmonary disease (COPD) [7], cancer [8], and cerebrovascular diseases [11] were all associated with worse COVID-19 health outcomes including death [5,6,7,11,12,13]. Furthermore, individuals with preexisting comorbidities might have had less opportunity to obtain the required healthcare during the pandemic as healthcare facilities were overwhelmed with COVID-19 cases [14].

It is important to highlight the significant regional variation in the prevalence of comorbidities across different regions of the United States. For instance, the southeast region exhibited a higher prevalence of diabetes and heart disease as compared to other US regions [15]. Specifically, cardiovascular diseases, including stroke, were found to be 34% more prevalent in the stroke belt, which encompasses 11 states in the southern part of the country [15]. Consequently, it is crucial to explore county-level characteristics to effectively allocate resources and develop targeted policies at a local level.

However, the existing literature has not thoroughly investigated the burden and the risk factors associated with chronic disease morbidities using the COVID-19 Severe Health Risk Index (SHRI). The COVID-19 SHRI, a collaborative effort between PolicyMap and *The New York Times* [16], was employed for the first time in this context. It quantifies the relative risk of severe COVID-19 symptoms among a county’s population, specifically associated with underlying health conditions. These conditions, as identified by the Centers for Disease Control and Prevention (CDC), include Chronic Obstructive Pulmonary Disease (COPD), heart disease, high blood pressure, diabetes, and obesity [17].

Also, it is important to explore the predictors of the COVID-19 SHRI. Race and ethnicity have been recognized as significant predictors of chronic diseases, with the literature supporting disparities in health outcomes among different racial and ethnic groups [18,19]. Therefore, incorporating the racial/ethnic majority as a predictor of the COVID-19 SHRI is important for developing targeted and effective public health interventions. This can shed light on potential systemic barriers to healthcare access and socio-economic determinants affecting chronic disease prevalence.

The literature shows that incorporating other indicators like health access indicators including insurance status, the availability of Intensive Care Unit (ICU) beds, healthcare worker coverage, and preventable hospitalizations are important in predicting chronic disease burden [20,21,22,23]. Health access indicators are crucial to consider as they signify the access and capacity of the healthcare system; a shortage in these indicators may result in delayed or inadequate treatment for patients with chronic conditions, leading to adverse consequences. By considering health access indicators in predicting the COVID-19 SHRI, healthcare policymakers and providers can better assess the adequacy of their resources and plan interventions to mitigate the burden of chronic diseases on the healthcare system.

Other indicators are also important to consider like internet access which has emerged as a significant social determinant of health. Internet access is a crucial determinant of health as it allows people to access telehealth services, which can be especially vital for those living in remote or underserved areas. Additionally, internet access facilitates social connections and support networks [24,25].

On the other hand, food insecurity is a well-established risk factor for many adverse health outcomes including cardiovascular diseases [26]. Although food insecurity was a public health problem during the pandemic, 1 in 9 households suffered from food insecurity prior to the pandemic in the US [27]. Food insecure individuals might rely on cheap and calorically dense diets rather than purchasing more expensive and nutritious diets [28,29]. Therefore, it is worth exploring the role of food insecurity as a predictor of the COVID-19 SHRI.

Air pollution is also needed to be included in predicting the COVID-19 SHRI as there are some regions in the US that are exposed to very high levels of particulate matter (PM_2.5_) like some counties in California and Texas with an average of (21 to 37) micrograms per cubic meter of air (μg/m^3^). These levels exceed the upper limit levels set by the WHO (<10 μg/m^3^) [30,31]. Studies have established a clear link between exposure to air pollutants and the development of chronic conditions, such as cardiovascular diseases and respiratory disorders [32,33]. By integrating air pollution as a predictor, policymakers and public health authorities can identify high-risk areas and vulnerable populations to mitigate the burden of chronic diseases related to air pollution.

Lastly, population health behaviors have been investigated as predictors of chronic diseases. Smoking is considered a major risk factor for many chronic diseases including cardiovascular diseases and cancer [34]. Other daily habits like insufficient sleep may predict chronic diseases [35]. However, there is a lack of studies that explore the relationship between insufficient sleep and chronic diseases on a population level. Therefore, health behaviors were also included in this study.

Exploring the predictors of the COVID-19 SHRI would provide valuable insights into the counties in the United States that are already overwhelmed with the dual challenges of comorbidities and the pandemic. This approach can assist public health officials in directing appropriate resources to areas with a high burden of disease during the times of public health crisis. Therefore, the objectives of this study were (1) to investigate potential population risk factors preceding the COVID-19 pandemic that are associated with the COVID-19 SHRI utilizing non-spatial regression models and (2) to evaluate the performance of spatial regression models in comparison to non-spatial regression models. These objectives were assessed using counties major racial/ethnic groups, rurality, socioeconomics, health access, and environmental risk factors. The selection of variables for this research was based on indicators reported in peer-reviewed studies.

## 2. Materials and Methods

### 2.1. Dependent Variable

In this study, we utilized the COVID-19 SHRI to identify counties with high rates of underlying conditions that significantly increased the risk of severe COVID-19 infections within their communities. The COVID-19 SHRI is a composite index of the conditions that are associated with worse COVID-19 outcomes including Chronic Obstructive Pulmonary Disease (COPD), heart disease, high blood pressure, diabetes, and obesity [16,17].

A county’s COVID-19 SHRI score was calculated based on the sum of the estimated number of people ever diagnosed with each health condition. Subsequently, the scores were normalized according to the county’s populations to enable a direct comparison between counties with differing population sizes. It is important to note that the raw and normalized indices should not be interpreted as an accurate representation of the absolute number or percentage of individuals affected by the five conditions, as these shares are not mutually exclusive. For instance, individuals diagnosed with two or more conditions were counted multiple times [16,17].

### 2.2. Independent Variable

**Figure 1:**
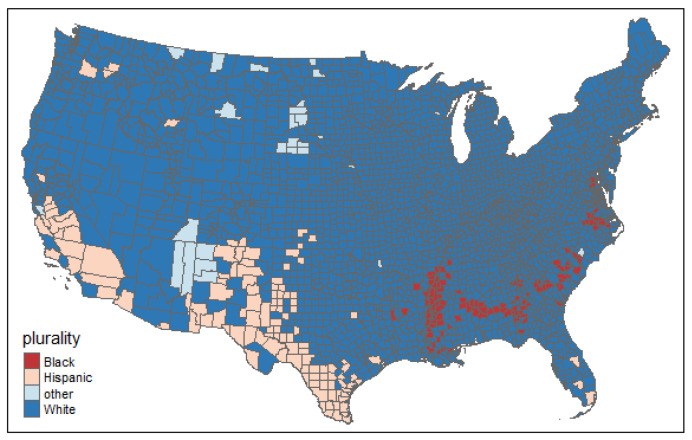
The spatial distribution of major racial/ethnic groups.

The primary independent variable in this study was constructed using the methodology employed by the census bureau to create a racial/ethnic majority variable per US county [36]. We utilized this approach to determine the racial/ethnic majority within each county. The following groups were considered in the racial/ethnic majority calculations: Hispanic, White (non-Hispanic), Black (non-Hispanic), and Other (including American Indian and Alaska Native non-Hispanic, Asian non-Hispanic, Native Hawaiian, and Other Pacific Islander non-Hispanic). Based on the predominant racial group within a county, as determined by analyzing the percentage of the population belonging to the largest racial/ethnic group, each county was assigned to one of the aforementioned racial/ethnic categories. Data regarding the total population and different racial/ethnic groups per county were obtained from the American Community Surveys 5-year estimates (ACS) [37]. Figure 1 shows the distribution of this variable.

### 2.3. Covariates

Table 1 provides the variable definitions, units of measure, and sources. As covariates in our analysis, we included the percentage of rurality as a continuous variable, which was derived from the County Health Ranking and Roadmaps [38]. By utilizing this continuous variable, our study provided a more detailed categorization of rurality that avoids the limitations associated with categorical variables that categorize rurality/urbanity into a few broad levels. This approach is crucial as it prevents the introduction of a false sense of immunity in rural counties. While previous research often employed binary descriptions of metropolitan versus non-metropolitan counties in health outcomes research [39,40], only a limited number of studies have employed a more granular description based on the three levels of metropolitan, micropolitan, and rural areas [41,42]. Furthermore, only a few studies have utilized a continuous variable to measure rurality, which we included in our study (see Table 1).

In addition, various sociodemographic variables were incorporated, including median age, income, men to women ratio, and high school completion rate. These data were obtained from the American Community Surveys 5-year estimates (ACS) [37]. The health indicators considered in this study encompassed smoking, insufficient sleep, Intensive Care Unit (ICU) beds, health insurance, Primary Care Providers (PCP), and preventable hospitalizations. Other variables included food insecurity, internet access, and air pollution resulting from particulate matter (PM_2.5_). Data for all these variables were sourced from County Health Ranking and Roadmaps [43] and PolicyMap [44]. The variables included in this study pertain to county-level data from 3107 counties within the continental United States. To analyze the data, a US counties shape file from the “Urban Map” package in the R environment was used for data projection [45] (See Table 1).

### 2.4. Data Analysis

The data analysis consisted of two stages. In the first stage, a descriptive summary was provided for continuous variables, stratified according to the four racial/ethnic majority variables and the overall sample. Mean values with standard deviations are presented for these variables. One-way Analysis of Variance (ANOVA) was employed to assess the significance of differences among the racial/ethnic majority groups. Normal distribution of all variables included in the study was verified.

In the second stage, multiple linear regression (MLR) models were utilized to investigate the association between major racial/ethnic group, other covariates, and the COVID-19 SHRI. The MLR models used the COVID-19 SHRI per county as the outcome variable of interest, which was continuous. The regression models’ coefficients (B), associated confidence intervals (CIs) and *p*-values are reported. A *p*-value < 0.05 indicated statistical significance. To address multicollinearity, the variation inflation factor (VIF) was assessed for all variables, and any factor with a VIF greater than 5 was removed from the model. The assumptions of linearity, constant variance, and normality were evaluated to diagnose the regression model.

However, it should be noted that the MLR models do not consider spatial aspects. Therefore, it may not be the most appropriate approach [32,33]. If spatial autocorrelation exists, MLR might lead to inflated model precision and type I errors. Additionally, if there is spatial dependence, the assumption of independent observations is violated. Hence, to account for spatial dependence, spatial analysis was used as an alternative approach. Before employing spatial modeling, the data were tested for spatial dependencies. The global Moran’s I of the COVID-19 SHRI and the global Moran’s I of the linear regression residuals were calculated to assess spatial correlation. The weight matrix was generated using the “Queen’s contingency” method, which is commonly employed in US county-level research [34].

The decision to proceed with spatial analyses was based on the results of Moran’s I for the COVID-19 SHRI and the Moran’s I for the linear regression residuals. As both Moran’s I results were significant (Moran’s I = 0.64, *p*-value < 0.05 for COVID-19 SHRI; Moran’s I = 0.22, *p*-value < 0.05 for residuals), we determined that it was appropriate to conduct spatial analyses and construct both the Spatial Lag model (SLM) and Spatial Error model (SEM).

SLM reveals a connection between the outcomes in one spatial unit and those in another [46]. It implies that the outcome variable in location a is influenced by the neighboring location b [47,48].

The formula for the SLM is:

Y=Xβ+ρWY+ε.

Y = dependent variable.

ρ = lag coefficient.

W = spatial weight matrix.

β = coefficient for a vector of neighborhood contexts.

ε = error term.

The SEM implies that the error term in location a is influenced by the neighboring location b [46,47,48].

The formula for the SEM is:

Y=Xβ+λWε+u.

Y: dependent variable.

β: coefficient for a vector of neighborhood contexts.

λ: coefficient.

W: spatial weight matrix.

ε: residual error matrix.

u: the normal assumption for the error term.

To assess the performance and fitness of the regression models, several model fitness statistics were calculated. A comparison was made between the results of the following tests: Akaike Information Criterion (AIC), Bayesian Information Criterion (BIC), log likelihood, and Moran’s I of the residuals. These measures were employed to determine the best model fit [49].

**Table 1:** Study variable descriptions, analytical roles, types, and sources.

Variable Name	Variable Description	Analytical Role	Variable Type	Source
COVID-19 SHRI	Severe COVID-19 health risk index in 2020	Dependent Variable	Continuous	[16]
Majority Racial/Ethnic Group	County’s predominant racial or ethnic group	Predictor	Categorical 1 = Majority Non-Hispanic White 2 = Majority Non-Hispanic Black 3 = Majority Hispanic 4 = Majority Non-Hispanic Other	Calculated from census row data. American Community Surveys 5-year estimates (ACS) 2020 [37]
Men/Women Ratio	Estimated ratio of men to women (number of males per hundred females), between 2015 and 2019 by county in United States	Covariate	Continuous	American Community Surveys 5-year estimates (ACS) 2020 [37]
Rural	% Rural: census population estimates	Covariate	Continuous	County health ranking [38]
Completed High School	Estimated percent of people with at least a high school diploma, between 2015 and 2019 by County in United States	Covariate	Continuous	American Community Surveys 5 years estimates (ACS) 2020 [37]
Age	Estimated median age of all people, between 2015 and 2019 by county in United States	Covariate	Continuous	American Community Surveys 5 years estimates (ACS) 2020 [37]
Smoking	Estimated percent of adults reporting to have ever smoked cigarettes in 2018 by county in United States	Covariate	Continuous	PolicyMap [44] 2017 and 2018 CDC Behavioral Risk Factor Surveillance System
Insufficient Sleep	Percentage of adults who reported fewer than 7 h of sleep on average (age-adjusted)	Covariate	Continuous	County Health Ranking [38]
ICU Beds	Number of Intensive Care Unit (ICU) beds in 2019 by county in United States	Covariate	Continuous	PolicyMap [44] Kaiser Health News
Primary Care Physicians (PCP)	Number of primary care doctors per 10,000 residents	Covariate	Continuous	Health Resources & Services Administration. Area health resource files [50]
Uninsured Population	Estimated percent of all people without health insurance, between 2015 and 2019 by county in United States	Covariate	Continuous	American Community Surveys 5-year estimates (ACS) 2020 [37]
Preventable Hospitalization	Rate of hospital stays for ambulatory-care-sensitive conditions per 100,000 Medicare enrollees	Covariate	Continuous	County health ranking [38]
HH with Internet Access	Estimated percent of households with internet access, between 2015 and 2019 by county in United States	Covariate	Continuous	American Community Surveys 5-year estimates (ACS) 2020 [37]
Food Insecurity	Estimated food insecurity rate in 2017 by county in United States	Covariate	Continuous	PolicyMap [44] Feeding America
PM_2.5_	Levels of PM_2.5_ per county	Covariate	Continuous	EPA [51]

## 3. Results

### 3.1. Descriptive Analysis

Table 2 provides a descriptive analysis of the study variables (*n* = 3107), categorized by the four levels of racial/ethnic majority and the overall sample. The average COVID-19 SHRI varied among the racial/ethnic majority levels, with majority Black counties exhibiting the highest burden (108.65 ± 12.90). Socioeconomic indicators also exhibited variations among the racial/ethnic majority levels. Notably, majority Black counties had the lowest average household income (43,411.35 ± 11,550.58) compared to the national average (57,610.36 ± 14,586.42), along with the lowest percentage of high school graduates (76.08 ± 8.97) compared to the national average (87.64 ± 6.01). Additionally, majority minority counties had a younger population compared to the national average.

Regarding health behavior and health access indicators, the average rate of current adult smokers was (20.31 ± 4.21), with higher rates observed in majority other racial/ethnic counties. The average rate of insufficient sleep was (36.75 ± 3.96), with the highest prevalence in majority Black counties (42.76 ± 2.28) compared to the national average (36.80 ± 3.96).

In terms of health access indicators, the average number of primary care providers per county’s population was (54.91 ± 36.48). The average rate of preventable hospitalizations was higher in majority Black counties (5082.67 ± 1412.71) compared to the national average (4037.70 ± 1542.13). The average logarithm of national ICU beds was (0.61 ± 0.43), and the average uninsured population per county was (9.55 ± 5.09), with a higher proportion of uninsured individuals in majority minority counties compared to the national average.

Environmental indicators were also considered in the study. The average concentration of PM_2.5_, representing air pollution, was (8.03 ± 1.70). The average population with broadband access was (78.64 ± 8.23), with majority Black counties having lower internet coverage compared to the national average (69.21 ± 11.04). A similar trend was observed for food insecurity, with a national average of (13.24 ± 3.95) compared to an average of (22.47 ± 4.61) in the majority of Black counties (See Table 2).

### 3.2. Regression Analyses Results

The results of the fully adjusted non-spatial and spatial regression analyses predicting the COVID-19 SHRI are presented in Table 3. Further detailed analyses of the partially adjusted models are provided in Appendix A. The stepwise adjusted regression models were constructed as Model 1 that adjusted for the sociodemographic factors followed by Model 2 that adjusted for the health behavior and health access indicators. Model 3 adjusted for the variables mentioned in Model 2 plus adjusting for the environmental indicators (see Appendix A and Table 3).

Majority racial/ethnic groups and other counties’ sociodemographic characteristics, health and health access indicators, and environmental indicators demonstrated robust associations with the COVID-19 SHRI across both spatial and non-spatial regression models. Notably, racial/ethnic majority played a significant role, with majority White [MLR (β = −4.53, 95% CI = −6.14, −2.92), SLM (β = −4.62, 95% CI = −6.04, −3.19), SEM (β = −3.65, 95% CI = −5.18, −2.11)] and majority Hispanic [MLR (β = −6.45, 95% CI = −8.82, −4.07), SLM (β = −4.76, 95% CI = −6.86, −2.66), SEM (β = −3.08, 95% CI = −5.43, −0.73)] exhibiting lower COVID-19 SHRI scores compared to majority Black counties. Additionally, counties with an older age population had a higher burden of high COVID-19 SHRI scores [MLR (β = 0.52, 95% CI = 0.45, 0.59), SLM (β = 0.56, 95% CI = 0.50, 0.62), SEM (β = 0.71, 95% CI = 0.65, 0.78)]. Higher household income was associated with lower COVID-19 SHRI scores. For more details about the associations, refer to Table 3.

Moving on to health behaviors and health access indicators, higher rates of smokers [MLR (β = 0.82, 95% CI = 0.68, 0.97), SLM (β = 0.56, 95% CI = 0.43, 0.69), SEM (β = 0.96, 95% CI = 0.80, 1.13)], populations reporting insufficient sleep [MLR (β = 0.78, 95% CI = 0.67, 0.90), SLM (β = 0.46, 95% CI = 0.35, 0.56), SEM (β = 0.83, 95% CI = 0.68, 0.97)], and preventable hospitalizations [MLR (β = 4.17, 95% CI = 3.35, 4.99), SLM (β = 2.78, 95% CI = 2.04, 3.51), SEM (β = 2.66, 95% CI = 1.85, 3.48)] were associated with higher COVID-19 SHRI scores. Conversely, counties with greater coverage of primary care providers (PCP) and ICU beds demonstrated lower COVID-19 SHRI scores. Environmental indicators also revealed significant associations, with higher levels of PM_2.5_ concentration (environmental pollution) associated with higher COVID-19 SHRI scores [MLR (β = 0.75, 95% CI = 0.56, 0.94), SLM (β = 0.46, 95% CI = 0.29, 0.63), SEM (β = 0.53, 95% CI = 0.28, 0.78)]. Counties with higher rates of internet access were associated with a lower COVID-19 SHRI scores [MLR (β = −0.13, 95% CI = −0.19, −0.07), SLM (β = −0.09, 95% CI = −0.15, −0.04), SEM (β = −0.07, 95% CI = −0.13, −0.02)] (see Table 3).

The COVID-19 SHRI, measured on a scale of 0 to 100, served as the continuous outcome variable for this study. The fully adjusted linear regression model accounted for 71% of the variance in the COVID-19 SHRI. Model assumptions were assessed, and the goodness-of-fit was evaluated using the Hosmer-slow statistic (*p* > 0.05). All included variables exhibited low VIF factors (<5).

**Figure 2:**
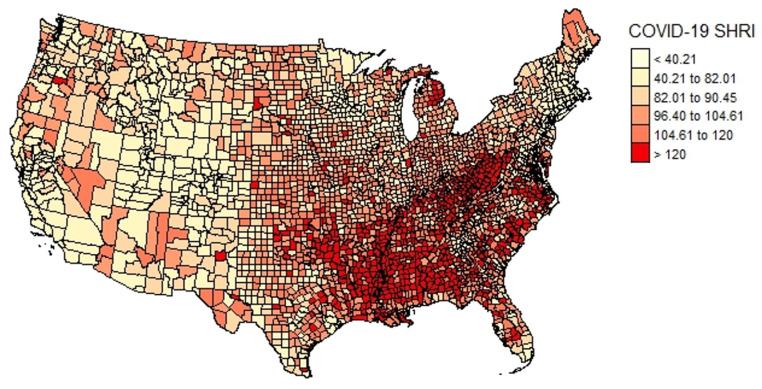
The spatial distribution of COVID-19 SHRI (Moran’s I = 0.63, *p*-value < 0.05).

**Figure 3:**
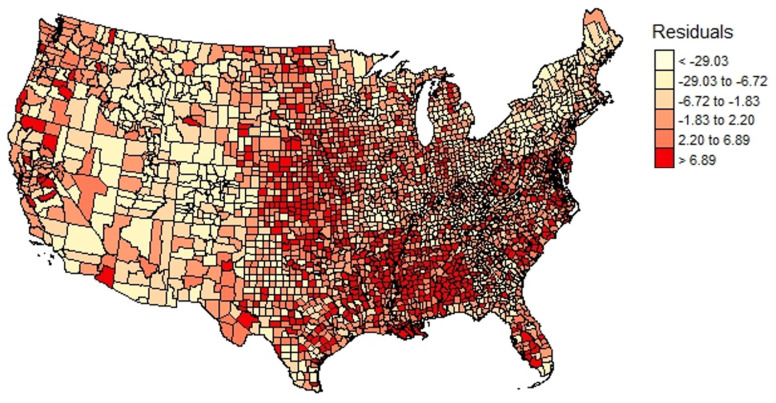
The spatial distribution of COVID-19 SHRI linear regression analysis residuals (Moran’s I = 0.22, *p* value <0.05).

The AIC, BIC, log likelihood, and Moran’s I of the residuals were analyzed. AIC and BIC slightly favored the Spatial Lag and Spatial Error models over the multiple linear regression model. The spatial error model had a Lambda (λ) of 0.60 (95% CI 0.57, 0.64), and the Spatial Lag model had a Rho (ρ) of 0.41 (95% CI 0.38, 0.44). The Moran’s I of the residuals was significant (*p* < 0.05) for the linear regression model. The goodness-of-fit parameters indicated that the spatial regression analyses provided added significance beyond what the linear regression analyses demonstrated, although the differences were minimal (see Table 3 and Figure 2 and Figure 3).

## 4. Discussion

This study sought to (1) investigate potential population risk factors preceding the COVID-19 pandemic that are associated with the COVID-19 SHRI utilizing non-spatial regression models and (2) evaluate the performance of spatial regression models in comparison to non-spatial regression models. The objectives were assessed by including the major racial/ethnic groups, rurality, socioeconomics, health access, and environmental risk factors. The findings provided a comprehensive understanding of these objectives and highlighted the importance of investigating the burden of high COVID-19 SHRI scores as a preliminary step in identifying populations at high risk. The study introduced the COVID-19 SHRI as a valuable population-level index for predicting adverse COVID-19 outcomes, with potential applicability in future pandemics. The results can inform state health departments and local partners for effective surveillance, program planning, and resource allocation to address chronic diseases.

Both the non-spatial and spatial regression models showed some consistent associations between the variables included in the study and the COVID-19 SHRI; the key associations observed were racial/ethnic majority, age, household income, health behaviors, health access, and environmental factors with the COVID-19 SHRI.

This study found that majority White and majority Hispanic counties had lower COVID-19 SHRI scores compared to majority Blacks counties. This finding aligns with previous research showing a higher prevalence of chronic disease clustering in Black populations [18,19]. Most of the counties with higher COVID-19 SHRI scores were clustered in the southeast region of the US. This study contributes to the literature by highlighting the role of racial/ethnic disparity in shaping ecological health outcomes among different US populations. It also reveals disproportionate distributions of social, economic, and health indicators in majority Black counties, paving the way for future research to explore other health outcomes using the racial/ethnic majority typology.

Counties with higher average rurality had a greater COVID-19 SHRI scores. The existing literature has already investigated rural disparities in health outcomes, which are characterized by older populations, higher comorbidity rates, and limited access to healthcare [52,53]. This study’s findings highlight the importance of conducting more detailed analyses in rural regions to explore the clustering of higher health risk factors contributing to a worse COVID-19 SHRI and overall health outcomes. Therefore, future research should further investigate the role of rurality in predicting population health outcomes.

Counties with a larger older population had higher COVID-19 SHRI scores, aligning with previous literature [19]. This association can be attributed to biological deficits that occur with aging, emphasizing the need for public health measures to improve access to healthcare in counties with a higher proportion of elderly residents. Conversely, a higher male to female ratio was associated with lower COVID-19 SHRI scores. However, the literature has inconsistent results regarding the prevalence of multiple chronic conditions in males versus females [18,54]. Further research is necessary to investigate the association between gender and the burden of comorbidities.

Higher household income was associated with lower COVID-19 SHRI scores, likely due to improved healthcare access and healthier lifestyle choices in affluent counties [19]. Certain health behavior indicators showed significant positive associations with the COVID-19 SHRI. Counties with higher percentages of smokers and individuals with insufficient sleep had higher COVID-19 SHRI scores. Smoking is a well-known contributor to chronic diseases such as coronary heart disease, COPD, and cancer, underscoring the importance of targeted prevention intervention [55]. Insufficient sleep affects the health and quality of life for around 70 million Americans, and its negative consequences have been documented in the literature. Addressing insufficient sleep aligns with the Healthy People 2030 goal of increasing the percentage of people obtaining sufficient sleep [56].

Some health access indicators were significantly associated with the COVID-19 SHRI. Counties with a higher percentage of primary care physicians (PCP) and intensive care unit (ICU) beds showed lower COVID-19 SHRI scores. This finding is important considering that two-thirds of emergency room visits in the US are attributed to chronic conditions, adding to the cost and strain on healthcare systems [57]. Additionally, this study revealed that counties with high rates of preventable hospitalizations had higher COVID-19 SHRI scores, highlighting the importance of timely access to routine medical visits and primary care providers to prevent such hospitalizations [58]. Ensuring better healthcare access opportunities and an adequate healthcare workforce are crucial for promoting healthier populations.

The study also included environmental indicators, such as particulate matter (PM_2.5_), known to be hazardous to health. Consistent with previous research, higher levels of PM_2.5_ were associated with higher COVID-19 SHRI scores. For example, Bennett et al. conducted a study analyzing vital statistics data from 1999 to 2015, which consistently demonstrated higher death rates associated with elevated concentrations of PM_2.5_ over time for both males and females [59]. These findings underscore the importance of public health policies aimed at controlling high levels of PM_2.5_ to protect public health. Conversely, access to the internet showed the opposite pattern. This study revealed that counties with higher internet access had lower COVID-19 SHRI scores. Adequate internet access is recognized as an important social determinant of health, facilitating better communication between healthcare providers and patients through telehealth and the use of remote monitoring devices [24]. Thus, the association between higher internet access and lower COVID-19 SHRI scores aligns with expectations.

This study has certain limitations that should be considered. First, the analyses conducted were ecological in nature, which means they did not examine individual-level risk factors for the COVID-19 SHRI. However, the primary focus of this study was to identify county-level characteristics of at-risk counties in order to inform resource allocation efforts, rather than estimating individual-level characteristics. Second, the study utilized covariates from different years. Although this approach may raise concerns, it is important to note that previous studies have utilized this methodology, as the data being described pertains to populations rather than individuals [60,61].

## 5. Conclusions

The study used county data to explore the potential population risk factors that were associated with the COVID-19 SHRI using spatial and non-spatial regression models, revealing distinct patterns of burden. The findings underscored the importance of place and race/ethnicity as determinants of health outcomes, highlighting the need for targeted resource allocation and interventions to address social determinants of health, particularly among vulnerable racial minority groups. The spatial models outperformed the non-spatial models, serving as a foundation for future spatial modeling of population-level health outcomes. This study emphasized the potential for modifying socioeconomic and health-related factors such as access to quality healthcare, evidence-based interventions, and economic opportunities.

## Figures and Tables

**Table 2:** Study variables according to major racial/ethnic group and the overall sample (*n* = 3107) by US county.

Characteristics	Majority White 2826	Majority Black 130	Majority Hispanic 125	Majority Other 27	Overall Sample	*p*-Value
COVID-19 SHRI	92.59 ± 12.96	108.65 ± 12.90	83.70 ± 9.60	98.23 ± 11.54	92.85 ± 13.37	<0.01
% Rural	59.68 ± 30.89	52.59 ± 34.40	35.09 ± 30.70	76.64 ± 27.06	58.06 ± 31.55	<0.01
Household Income	58,169.34 ± 14,246.66	43,411.35 ± 11,550.58	54,951.38 ± 12,075.17	43,555.07 ± 20,318.78	57,610.36 ± 14,586.42	<0.01
Age	41.95 ± 5.21	39.25 ± 3.91	35.83 ± 5.29	31.89 ± 3.64	41.47 ± 5.36	<0.01
Completed High School	88.36 ± 5.21	81.92 ± 5.28	76.08 ± 8.97	84.44 ± 4.46	87.64 ± 6.01	<0.01
Men to Women Ratio	100.62 ± 10.45	98.65 ± 18.79	105.35 ± 13.60	98.66 ± 2.76	100.67 ± 11.17	<0.01
Smoking	20.31 ± 4.02	23.39 ± 3.33	16.5449 ± 2.2915	28.4630 ± 6.4718	20.3097 ± 4.2103	<0.01
Insufficient Sleep	36.57 ± 3.88	42.76 ± 2.28	36.2595 ± 2.1045	37.8013 ± 2.7749	36.7958 ± 3.9591	<0.01
Log ICU Beds	0.67 ± 0.52	0.64 ± 0.58	0.74 ± 0.69	0.30 ± 0.28	0.61 ± 0.43	<0.01
% Uninsured	9.01 ± 4.42	12.02 ± 3.11	16.13 ± 6.97	23.57 ± 8.84	9.55 ± 5.09	<0.01
% PCP	54.88 ± 36.44	48.01 ± 31.51	42.75 ± 22.71	49.99 ± 24.80	54.91 ± 36.48	<0.01
Preventable Hospitalizations	4000.22 ± 1531.34	5082.67 ± 1412.71	3996.67 ± 1454.46	4605.11 ± 2099.23	4037.70 ± 1542.14	<0.01
PM_2.5_	7.98 ± 1.56	8.61 ± 0.89	8.76 ± 3.05	6.24 ± 1.96	8.01 ± 1.65	<0.01
Internet Access	79.27 ± 7.47	69.21 ± 11.04	75.06 ± 10.06	63.10 ± 11.64	78.64 ± 8.23	<0.01
Food Insecurity	12.83 ± 3.33	22.47 ± 4.61	11.28 ± 2.76	19.51 ± 3.55	13.24 ± 3.95	<0.01

**Table 3:** Multiple linear regression (MLR), Spatial lag (SLM), and Spatial Error (SEM) modeling COVID-19 SHRI in continental United States (*n* = 3107).

Characteristics	MLR			SLM			SEM		
		95% CI			95% CI			95% CI	
	β	Lower	Upper	β	Lower	Upper	β	Lower	Upper
Major Racial/Ethnic Group (Ref = Majority Black)									
Majority Hispanic	−6.45 ***	−8.82	−4.07	−4.76 ***	−6.86	−2.66	−3.08 **	−5.43	−0.73
Majority Other	−5.85 **	−9.74	−1.97	−0.13	−3.59	3.33	−1.92	−5.51	1.66
Majority White	−4.53 ***	−6.14	−2.92	−4.62 ***	−6.04	−3.19	−3.65 ***	−5.18	−2.11
Rural	0.03 ***	0.02	0.05	0.02 ***	0.01	0.03	0.01 **	0.00	0.02
Log Household Income	−4.71 ***	−7.16	−2.26	−2.57 **	−4.74	−0.41	−3.49 **	−5.93	−1.06
Age	0.52 ***	0.45	0.59	0.56 ***	0.50	0.62	0.71 ***	0.65	0.78
Completed High School	−0.04	−0.12	0.04	−0.07	−0.14	0.00	−0.04	−0.13	0.04
Men to Women Ratio	−0.15 ***	−0.18	−0.12	−0.11 ***	−0.14	−0.09	−0.10 ***	−0.13	−0.08
Smokers	0.82 ***	0.68	0.97	0.56 ***	0.43	0.69	0.96 ***	0.80	1.13
Insufficient Sleep	0.78 ***	0.67	0.90	0.46 ***	0.35	0.56	0.83 ***	0.68	0.97
Log ICU Beds	−0.01 ***	−0.02	−0.01	−0.01 ***	−0.01	−0.01	−0.01 ***	−0.01	−0.01
Uninsured	0.01	−0.06	0.08	0.05	−0.02	0.12	0.05	−0.03	0.14
PCP	−0.02 ***	−0.03	−0.02	−0.03 ***	−0.04	−0.02	−0.01 ***	−0.02	−0.01
Log Preventable Hospitalization	4.17 ***	3.35	4.99	2.78 ***	2.04	3.51	2.66 ***	1.85	3.48
PM_2.5_	0.75 ***	0.56	0.94	0.46 ***	0.29	0.63	0.53 ***	0.28	0.78
Broadband	−0.13 ***	−0.19	−0.07	−0.09 ***	−0.15	−0.04	−0.07 **	−0.13	−0.02
Food Insecurity	0.16 **	0.03	0.28	0.03	−0.08	0.14	−0.07	−0.21	0.08
Goodness of Fit									
Rho				0.41	0.38	0.44			
Lambda							0.60	0.57	0.64
AIC	20,039.82			19,453			19,377		
Log Likelihood	−10,000.91			−9706.53			−9668.26		
BIC	20,153.46			19,203.51			19,496.11		
Moran’s I of the Residuals	0.22 **			0.08			−0.022		

Note: ‘***’ means *p*-value < 0.001, ‘**’ means *p*-value < 0.01.

## Data Availability

Data were obtained from Publicly available sources. Please refer to Table 1.

## Appendix A

**Table A1:** Multiple linear regression table modeling COVID-SHRI in continental United States (*n* = 3107 counties).

	Model 1				Model 2				Model 3			
Characteristics	β	95% CI		VIF	β	95% CI		VIF	β	95% CI		VIF
		Lower	Upper			Lower	Upper			Lower	Upper	
Major Racial/Ethnic Group (Ref = Majority Black				1.72				2.21				3.14
Majority Hispanic	−9.80 ***	−2.18	−17.41		−7.82 ***	−9.97	−5.68		−6.45 ***	−8.82	−4.07	
Majority Other	−6.82 ***	−10.81	−2.84		−7.40 ***	−11.29	−3.50		−5.85 **	−9.74	−1.97	
Majority White	−6.41 ***	−8.12	−4.69		−5.78 ***	−7.27	−4.29		−4.53 ***	−6.14	−2.92	
Rural	0.05 ***	0.03	0.06	1.62	0.04 ***	0.02	0.05	2.45	0.03 ***	0.02	0.05	2.42
Log Household Income	−22.56 **	−24.40	−20.71	1.58	−7.91 ***	−9.83	−6.00	2.01	−4.71 ***	−7.16	−2.26	3.58
Age	0.44 ***	0.36	0.51	1.48	0.50 ***	0.43	0.56	2.20	0.52 ***	0.45	0.59	2.20
Completed High School	−0.65	−0.72	−0.58	1.77	−0.11 *	−0.19	−0.03	2.92	−0.04	−0.12	0.04	3.17
Men to Women Ratio	−0.18 ***	−0.21	−0.15	1.07	−0.16 ***	−0.19	−0.13	1.11	−0.15 ***	−0.18	−0.12	1.12
Smokers					0.89 ***	0.75	1.03	4.74	0.82 ***	0.68	0.97	4.80
Insufficient Sleep					0.79 ***	0.67	0.90	3.30	0.78 ***	0.67	0.90	3.36
Log ICU Beds					−0.01 ***	−0.01	−0.01	1.28	−0.01 ***	−0.02	−0.01	1.32
Uninsured					0.03	−0.04	0.10	1.70	0.01	−0.06	0.08	1.76
PCP					−0.03 ***	−0.04	−0.02	1.38	−0.02 ***	−0.03	−0.02	1.41
Log Preventable Hospitalization					4.50 ***	3.67	5.34	1.48	4.17 ***	3.35	4.99	1.49
PM_2.5_									0.75 ***	0.56	0.94	1.31
Broadband									−0.13 ***	−0.19	−0.07	2.96
Food Insecurity									0.16 **	0.03	0.28	3.30

Note: ‘***’ means *p*-value < 0.001, ‘**’ means *p*-value < 0.01, ‘*’ means *p*-value < 0.05.

**Table A2:** Spatial lag regression modeling COVID-SHRI in continental United States (*n* = 3107 counties).

	Model 1			Model 2			Model 3		
Characteristics	β	95% CI		β	95% CI		β	95% CI	
		Lower	Upper		Lower	Upper		Lower	Upper
Major Racial/Ethnic Group (Ref = Majority Black									
Majority Hispanic	−9.69 ***	−11.59	−7.78	−5.17 ***	−7.07	−3.28	−4.76 ***	−6.86	−2.66
Majority Other	2.68	−0.46	5.83	−0.36	−3.81	3.09	−0.13	−3.59	3.33
Majority White	−4.96 ***	−6.32	−3.61	−5.15 ***	−6.46	−3.84	−4.62 ***	−6.04	−3.19
Rural	0.04 ***	0.02	0.05	0.02 ***	0.01	0.03	0.02 ***	0.01	0.03
Log Household income	−9.68 ***	−11.28	−8.07	−3.52 ***	−5.23	−1.81	−2.57 **	−4.74	−0.41
Age	0.52 ***	0.46	0.58	0.55 ***	0.49	0.61	0.56 ***	0.50	0.62
Completed High School	−0.41 ***	−0.47	−0.35	−0.13 ***	−0.20	−0.06	−0.07	−0.14	0.00
Men to Women Ratio	−0.11 ***	−0.13	−0.08	−0.12 ***	−0.14	−0.09	−0.11 ***	−0.14	−0.09
Smokers				0.58 ***	0.45	0.71	0.56 ***	0.43	0.69
Insufficient Sleep				0.46 ***	0.35	0.56	0.46 ***	0.35	0.56
Log ICU Beds				−0.01 ***	−0.01	0.00	−0.01 ***	−0.01	−0.01
Uninsured				0.05	−0.01	0.11	0.05	−0.02	0.12
PCP				−0.03 ***	−0.04	−0.02	−0.03 ***	−0.04	−0.02
Log Preventable Hospitalization				2.94 ***	2.20	3.68	2.78 ***	2.04	3.51
PM_2.5_							0.46 ***	0.29	0.63
Broadband							−0.09 ***	−0.15	−0.04
Food Insecurity							0.03	−0.08	0.14

Note: ‘***’ means *p*-value < 0.001, ‘**’ means *p*-value < 0.01.

**Table A3:** Spatial error regression table modeling COVID-SHRI in continental United States (*n* = 3107 counties).

	Model 1			Model 2			Model 3		
Characteristics	B	95% CI		B	95% CI		B	95% CI	
		Lower	Upper		Lower	Upper		Lower	Upper
Major Racial/Ethnic Group (Ref = Majority Black									
Majority Hispanic	−5.53 ***	−7.83	−3.23	−2.59 **	−4.77	−0.41	−3.08 **	−5.43	−0.73
Majority Other	7.55 ***	4.16	10.95	−1.69	−5.27	1.89	−1.92	−5.51	1.66
Majority White	−4.42 ***	−5.96	−2.87	−3.53 ***	−4.98	−2.09	−3.65 ***	−5.18	−2.11
Rural	0.04 ***	0.02	0.05	0.01	0.00	0.03	0.01	0.00	0.02
Log Household Income	−15.53 ***	−17.41	−13.65	−3.20 **	−5.25	−1.14	−3.49 **	−5.93	−1.06
Age	0.70 ***	0.64	0.77	0.72 ***	0.66	0.79	0.71 ***	0.65	0.78
Completed High School	−0.34 ***	−0.42	−0.27	−0.09	−0.17	−0.01	−0.04	−0.13	0.04
Men to Women Ratio	−0.07 ***	−0.10	−0.05	−0.11 ***	−0.13	−0.08	−0.10 ***	−0.13	−0.08
Smokers		−7.83	−3.23	0.98 ***	0.82	1.15	0.96 ***	0.80	1.13
Insufficient Sleep				0.83 ***	0.69	0.98	0.83 ***	0.68	0.97
Log ICU Beds				−0.01 ***	−0.01	−0.01	−0.01 ***	−0.01	−0.01
Uninsured				0.05	−0.04	0.13	0.05	−0.03	0.14
PCP				−0.02 ***	−0.02	−0.01	−0.01 ***	−0.02	−0.01
Log Preventable Hospitalization				2.76 ***	1.95	3.58	2.66 ***	1.85	3.48
PM_2.5_							0.53 ***	0.28	0.78
Broadband							−0.07 **	−0.13	−0.02
Food Insecurity							−0.07	−0.21	0.08

Note: ‘***’ means *p*-value < 0.001, ‘**’ means *p*-value < 0.01.
